# Multiorgan paradoxical embolism consequent to acute pulmonary thromboembolism with patent foramen ovale: a case report

**DOI:** 10.4076/1757-1626-2-8358

**Published:** 2009-09-17

**Authors:** Giorgio Caretta, Debora Robba, Ivano Bonadei, Melissa Teli, Benedetta Fontanella, Enrico Vizzardi, Davide Farina, Riccardo Raddino, Livio Dei Cas

**Affiliations:** 1 Department of Cardiology, University of BresciaPiazzale Spedali Civili 1, 25124Italy; 2 Department of Radiology, University of BresciaPiazzale Spedali Civili 1, 25124Italy

## Abstract

Paradoxical embolism is defined as a systemic arterial embolism requiring the passage of a venous thrombus into the arterial circulatory system through a right-to-left shunt. It is a relatively rare phenomenon, representing about 2% of all cases of arterial embolism. We report a case of a 79-years-old woman admitted to hospital because of dyspnea and lower left limb pain. CT scan revealed multiple thrombi to kidney, lower limb and superior mesenteric artery during acute pulmonary embolism. Echocardiogram documented a patent foramen ovale with a right-to-left shunt. The patient was treated with thrombolytic therapy and heparin with progressive improvement of symptoms and resolution of pulmonary embolism and peripheral thrombosis. Patent foramen ovale closure was not performed because a life-long anticoagulation therapy was necessary, a tunnel-type patent foramen ovale may increases difficulty in realizing device implantation and there are no clear evidence-based guidelines to date addressing treatment in presence of a patent foramen ovale.

## Introduction

Paradoxical embolism (PDE) is defined as a systemic arterial embolism requiring the passage of a venous thrombus into the arterial circulatory system through a right-to-left shunt [1].

It is a relatively rare condition accounting of nearly 2% of systemic arterial emboli [2] Nevertheless it could lead to severe prognosis, with a reported rate of mortality in 21% of cases [3].

This condition can be related to an abnormal intracardiac communication. The most common cardiac defect associated with paradoxical embolism is the patent foramen ovale (PFO), which reaches a prevalence that goes from 27% to 35% in the normal population [4].

Under physiological conditions, PFO determines a small amount of left-to-right shunt without any hemodynamic significant changes. However, in case of increased right atrial pressure, the inversion of shunt from right-to-left can occur, leading to paradoxical embolism [5].

We reported the case of an acute pulmonary thromboembolism complicated by a multiorgan PDE associated with PFO.

## Case presentation

A 79-year-old Caucasian Italian female was admitted to our hospital for dyspnea and referred lower left limb pain. Her past medical history included systemic hypertension for ten years, type 2 Diabetes Mellitus and chronic obstructive pulmonary disease.

At the admission she was markedly dyspneic, pale and had a pulse rate of 120 beats per minute. The blood pressure was 125/95 mmHg. At pulmonary auscultation there was evidence of broncospasm without signs of congestion.

The lower left leg was warm but pulseless. The oxygen saturation was reduced at about 82-85% in room air. Examination of arterial blood gases revealed hypocapnic hypoxia (pO2 46 mmHg, pCO2 21 mmHg, pH 7.52) and the electrocardiogram showed right axis deviation and inverted T waves in the anterior leads (V1-V3).

The laboratory examinations showed significant leukocytosis (21300/mm^3^), increased blood glucose and serum creatinine levels (Cr. 2.4 mg/dL), with D-dimer levels moderately high (715 ng/ml).

**Figure 1.**
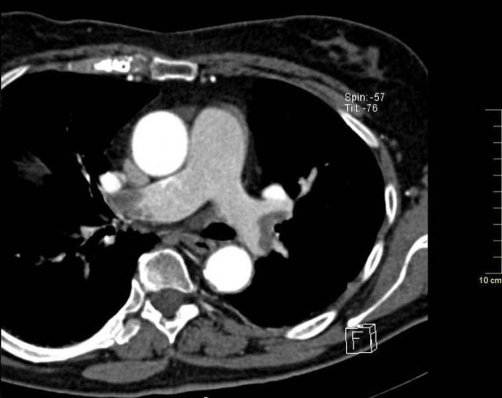
Contrast-enhanced CT image showing massive thrombosis of the right and left pulmonary artery (arrows).

**Figure 2.**
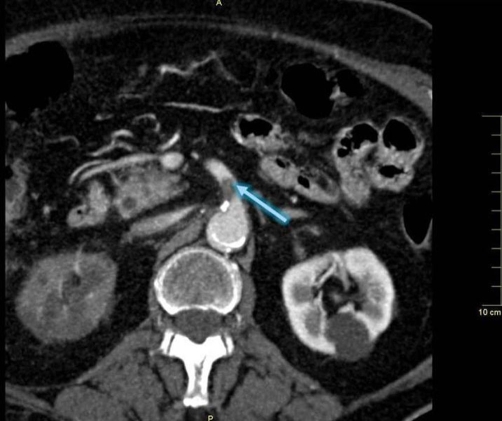
CT scan image showing thrombosis of the proximal portion of the superior mesenteric artery (arrow). A lack of contrast enhancement of the right kidney can also be appreciated.

**Figure 3.**
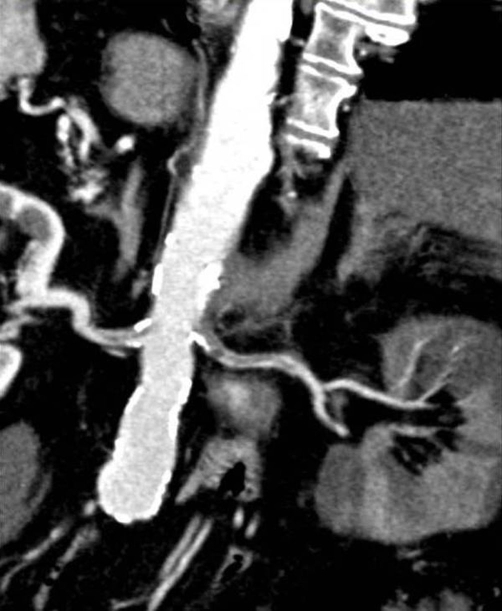
CT image of subocclusive thrombus of right renal artery (right arrow) showing renal hypoperfusion (diminished cortical contrast enhancement). Left arrow shows a floating trhrombus in left renal artery.

Echocardiography imaging revealed right ventricular dilatation and elevated transtricuspid systolic gradient with indirect signs of high pulmonary hypertension. The whole-body CT showed a massive thrombus in the main right and left pulmonary artery, extended to lobar branches (Figure 1). Moreover, the abdomen CT demonstrated the almost entirely occlusion of the superior mesenteric artery (Figure 2) and of the right renal artery, by a floating clot in the lumen, (Figure 3), resulting in renal hypoperfusion and further worsening of its function (Cr. 3.9 mg/dL). An embolic occlusion of the left external iliac artery was also evident. The Echo Doppler of her lower legs didn’t reveal signs of deep vein thrombosis, showing instead the whole thrombotic occlusion of poplytea artery. There was no evidence of ischemic lesions at the CT scan of brain.

**Figure 4.**
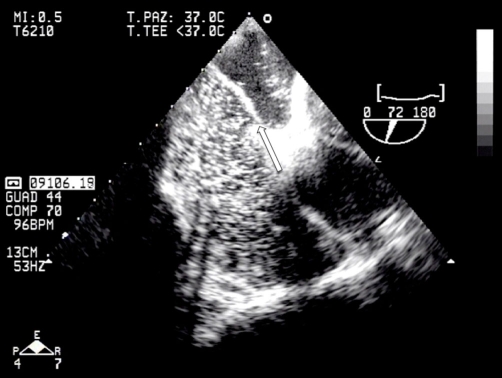
Transesophageal echocardiographic image of interatrial septum’s aneurysm with patent forame ovale.

Transesophageal echocardiography (TEE) excluded the presence of intracardiac thrombi but it revealed a significant interatrial septum’s aneurysm with long tunnel-type patent foramen ovale (PFO). The presence of other anatomic anomalies, frequently associated with patent foramen ovale [6], such as Chiari’s Network or a prominent Eustachian Valve were also excluded. A saline contrast echocardiography confirmed the presence of PFO and demonstrated a right-to-left shunt during Valsalva maneuver (Figure 4).

Because of the hemodynamic stability, the patient’s age and the absence of contraindications to thrombolisis, the woman was treated with i.v. tPA (10 mg bolus followed by 90 mg infusion over 2h) and i.v. heparin with progressive improvement of clinical conditions and dyspnea.

During the following days the kidney function gradually improves with a reduction of hematic creatinine from 3.9 to 1.3 mg/dL. A repeat CT one week later showed a partial resolution of pulmonary embolism and the reduction of renal, mesenteric and iliac thrombosis, while the left poplytea artery’s occlusion persisted.

Her anticoagulation work up showed no evidence of factor V Leiden or prothrombin gene mutation, antiphospholipid antibody screening was negative and level of homocysteine was normal. Levels of protein C, S and antithrombin III were unreliable in the context of an acute thromboembolic event or during antithrombotic therapy. Other predisposing factors for venous thromboembolism as metastatic solid tumors or lymphoproliferative disorders were excluded.

The patient was discharged 8 days after admission and no recurrent embolic episode was observed. She has to keep on assuming life-long oral anticoagulant therapy. PFO closure was not performed because our patient required a life-long anticoagulation therapy; the presence of a long tunnel-type PFO may give rise to technical problems in realizing device implantation and finally there are no clear guidelines based on randomized trials for therapy if patent foramen ovale is present.

## Discussion

First described by Cohnheim in 1877, PDE consists of the passage of embolic material through a right-to-left intracardiac shunt into the systemic circulation [7].

It is a relatively rare phenomenon, representing about 2% of all cases of arterial embolism. However, it is often associated to the diagnosis of PFO, estimating in about the 25-30% of normal population [2,3].

According to Johnson, the diagnostic criteria for PDE include:

1. A suitable source of embolic material in the venous circulation
2. Communication between systemic and pulmonary circulation of appropriate size, documented by an imaging test
3. Increased right heart pressure determining right-to-left shunting, in transient or longstanding [8,9].

We may presume all of these conditions occur in the reported case. In fact, deep vein thrombosis was not documented but the massive pulmonary embolism could imply its presence, a patency between right and left atrium was recognized and finally high pressure in the right atrium could be a consequence of the massive pulmonary embolism and also a right-to-left shunt was demonstrated during Valsalva maneuver with transesophageal echocardiography. A definitive diagnosis of PDE is possible only by means of an autotypic exam or by the evidence that a thrombus crosses through an intracardiac defect during echocardiography. However, paradoxical embolism can be presumed if the criteria of Johnson are satisfied or when arterial embolus and PFO are documented [5,10,11].

Transesophageal echocardiography may play a fundamental role in the diagnosis of PFO because capable of visualizing the interatrial septum, identifying and measuring abnormal intracardiac communications, it may reveal a right-to-left cardiac shunt or show intracardiac thrombus if present.

Multislice CT scan is used commonly to help diagnose pulmonary embolism or PDE, thanks to its high sensitivity, which is similar to that of pulmonary angiography. Pulmonary thromboembolism with PFO may be often complicated by PDE, especially if the patient is hemodynamically unstable.

The acute elevation of pulmonary mean artery pressure and also the increase in pulmonary resistance can promote an inversion shunting across the patent foramen ovale, leading to arterial embolism [5]. Transient right atrial hypertension due to acute pulmonary embolism can be the presumed mechanism for the occurrence of the simultaneous arterial embolism involving four organ systems such as in our case.

In many reported cases PDE may complicate pulmonary embolism [12,13], in one study it is estimated that 67% of cases are associated with pulmonary embolism. However, the multiple organs’ embolic involvement is less frequent: in the same study 23% of cases are described two different embolic sites and only 10% three different sites. Usual sites of PDE are the inferior limbs (49%), the brain (37%) and, more rarely, coronary, renal or splancnic arteries (4.5%) [14].

In order to prevent recurrent arterial emboli or paradoxical embolism, recommended treatment include observation, antiplatelet agents, systemic anticoagulation or closure of the PFO. According to many clinical cases, the different therapeutic options are not predictor of recurrence [15].

Patients with massive pulmonary embolism benefit from i.v. thrombolisis, which rapidly reverse the hemodynamic changes into the right-sided chambers [16].

A patient with presumptive hemodinamically significant PDE should be treated with thrombolytic therapy and heparin followed by long-lasting oral anticoagulation.

Intrapulmonary thrombolisis is indicated only in selected cases, when the drug should be concentrated more in a specific area, or in presence of contraindications to systemic therapy and additionally the concentrated dose may provide a faster resolution of the clot. However, our patient had the necessity of a systemic distribution of the drug [17].

The duration of anticoagulation therapy could be variable for the thrombotic risk, in example patients with moderate risk for venous thrombosis should take it for a period from 6 to 12 months. Patients at high risk for venous thrombosis may require a life-long anticoagulant therapy.

Inferior vena caval filter placement is not considered a first choice therapy for PDE because unable to trap small emboli (<3 mm) which may be asymptomatic in pulmonary circulation but may contribute to disastrous arterial occlusion in the systemic circulation [9].

The choice to close the PFO should also be individualized. The benefits should be weighed against the risks when choosing a treatment strategy [5,9]. Italian Stroke Guidelines (SPREAD) put oral anticoagulation and PFO closure at the same level [18]. In our case PFO closure was not performed because, in absence of a demonstrated source of emboli as cause of the massive embolism, there was the necessity of maintaining a long term anticoagulant treatment, the presence of a long tunnel-type PFO may give rise to technical problems in realizing device implantation and finally there are no clear guidelines based on randomized trials for therapy if patent foramen ovale is present. Such ongoing randomized studies could explain the efficacy of percutaneous closure as compared with medical therapy [19].

The inferior vena caval interruption or the closure of PFO may be considered as primary therapy in patients with contraindications for systemic anticoagulation [20].

## Conclusion

In conclusion, even if PDE is a quite rare phenomenon, PFO is relatively common in general population.

Therefore, signs of systemic embolism have to be carefully evaluated and recognized in presence of acute pulmonary embolism or deep venous thrombosis. In addition, sometimes clinical symptoms are not peculiar and it could be necessary consider the possible multiple organs’ involvement like in the reported case. Moreover, optimal treatment of PDE is controversial and it should be individualized.

## Authors’ contribuctions

GG performed the case and was a major contributor in writing the manuscript. DR, II, RR, LDC analyzed the literature about PDE and complications. BF and EV contributed to echocardiography examination and figures. DF contributed to CT examination and figures.

## Abbreviations

CT: computerized tomography
PDE: paradoxical embolism
PFO: patent foramen ovale
TEE: transesophageal echocardiography
tPA: tissue plasminogen activator
